# Radiotherapy integration after transoral robotic surgery in head and neck cancer: a bibliometric and knowledge-mapping analysis of adjuvant treatment, de-escalation, functional preservation, and survival outcomes

**DOI:** 10.3389/fonc.2026.1901904

**Published:** 2026-08-28

**Authors:** Defu Yang, Jianjing Wang, Ying Xu, Jing Liu, XinYing Ma, Ying Li, Dongyang Lv, Feng Shang

**Affiliations:** Department of Radiation Oncology, General Hospital of Northern Theater Command, Shenyang, China

**Keywords:** bibliometric analysis, de-escalation, functional preservation, head and neck cancer, HPV, knowledge mapping, oropharyngeal cancer, radiotherapy

## Abstract

**Background:**

Transoral robotic surgery (TORS) is increasingly considered within multimodality treatment pathways for selected head and neck cancers, in which postoperative pathology may influence radiotherapy (RT), chemoradiotherapy (CRT), treatment de-intensification, and functional preservation. This study characterized the development, knowledge structure, and thematic evolution of research on RT integration after TORS.

**Methods:**

Publications from 1 January 2010 to 1 August 2026 were retrieved from the Web of Science Core Collection, Scopus, and PubMed. After deduplication, exclusion of records lacking predefined fields required for downstream analyses, and manual topical screening, 215 publications were included. Bibliometric and knowledge-mapping analyses assessed publication and citation trends, contributors and collaboration, co-citation, bibliographic coupling, keyword and thematic evolution, and exploratory logistic life-cycle and latent Dirichlet allocation (LDA) models.

**Results:**

The 215 publications were distributed across 54 sources, with an annual growth rate of 14.72%; 60.0% were published during 2020–2026. International co-authorship accounted for 13.49%. The United States was the leading corresponding-author country, accounting for 96 publications (44.7% of the total corpus). Citation and co-citation structures linked foundational TORS studies with evidence on HPV-associated prognosis and postoperative RT/CRT. Keyword and thematic analyses showed persistent prominence of TORS and oropharyngeal squamous cell carcinoma, alongside increasing representation of adjuvant treatment, HPV-associated disease, swallowing, and functional outcomes. Bibliographic coupling and factorial analysis indicated substantial thematic overlap rather than clearly separated research domains. Exploratory logistic modeling showed only moderate fit (R² = 0.778) and did not fully capture the empirical publication peak, while the five-topic LDA solution had limited bootstrap stability (0.3356).

**Conclusion:**

The literature has shifted from predominantly procedure-centered investigation toward greater attention to risk-adapted postoperative management. Current research increasingly integrates treatment selection, RT dose and target-volume modification, and functional outcomes. Prospective multicenter studies with standardized postoperative risk definitions and long-term oncologic and functional assessment are needed to define the appropriate limits of treatment de-intensification.

## Introduction

Head and neck squamous cell carcinoma (HNSCC) comprises a biologically and clinically heterogeneous group of malignancies, among which oropharyngeal squamous cell carcinoma (OPSCC) has undergone a marked epidemiological shift with the increasing incidence of human papillomavirus (HPV)-associated disease (1, 2). HPV-positive OPSCC constitutes a distinct clinical subgroup with generally more favorable survival than HPV-negative disease (1, 3). This favorable prognosis has increased the importance of risk-adapted treatment strategies that maintain oncologic control while reducing treatment-related morbidity and preserving swallowing, speech, and long-term quality of life (1).

Radiotherapy (RT), alone or combined with systemic therapy, remains a major curative treatment for OPSCC (1). In the postoperative setting, treatment intensity is determined by pathological risk, and landmark randomized trials established the oncologic basis for adding concurrent chemotherapy to postoperative irradiation in selected patients with high-risk head and neck cancer (4, 5). In parallel, transoral robotic surgery (TORS) emerged as a minimally invasive surgical approach for selected oropharyngeal tumors, particularly tonsillar and base-of-tongue lesions, with early studies demonstrating technical feasibility and acceptable oncologic and functional outcomes (6–8). Importantly, TORS also provides pathological information—including margin status, nodal burden, and extranodal extension—that can modify recommendations for observation, postoperative RT, or postoperative chemoradiotherapy (CRT) (9, 10). TORS and RT are therefore increasingly considered components of a risk-adapted multimodality pathway rather than solely competing primary-treatment options.

Prospective studies have further defined this relationship. ORATOR directly compared primary RT with TORS plus neck dissection and pathology-directed adjuvant treatment, demonstrating excellent disease control with different toxicity and quality-of-life profiles; at final follow-up, swallowing-related quality-of-life scores converged, although modality-specific differences persisted in selected symptom domains (11, 12). ECOG-ACRIN E3311 subsequently evaluated pathology-guided postoperative de-intensification in resected p16-positive OPSCC, including reduced-dose RT for intermediate-risk disease, and reported favorable long-term disease control (13, 14). Subsequent swallowing analyses demonstrated functional recovery over time and early differences between the 50-Gy and 60-Gy postoperative RT groups, although these differences were not maintained at later follow-up (15). Complementary approaches include PATHOS, which evaluates risk-stratified reduction of postoperative treatment intensity after transoral surgery, and AVOID, which investigated omission of the resected primary-site bed from postoperative irradiation in carefully selected HPV-associated OPSCC (16, 17). Collectively, these studies have shifted attention from a binary comparison of TORS versus RT toward the integration of surgical selection, pathological risk stratification, chemotherapy use, RT dose and target-volume modification, and functional preservation.

The field has continued to develop toward greater standardization and evidence synthesis. Recent European surgical guidelines have addressed indications, contraindications, and perioperative practice for TORS in head and neck cancer (18). Contemporary systematic reviews and meta-analyses have compared TORS-based treatment with definitive RT or CRT (19) and examined oncologic and functional outcomes, pathological risk stratification, and treatment-de-escalation strategies in HPV-associated OPSCC (20). Although favorable outcomes have been reported in appropriately selected patients, substantial heterogeneity remains in patient selection, pathological risk definitions, adjuvant-treatment criteria, and treatment pathways. The clinically relevant question is therefore increasingly not whether TORS can replace RT, but how surgery and RT can be integrated to preserve oncologic control while minimizing avoidable multimodality treatment burden.

The expanding TORS literature has also prompted dedicated bibliometric analyses. Farasani et al. mapped TORS research from 2005 to 2025 and described a transition from technique-oriented investigation toward HPV-associated disease, functional outcomes, and treatment de-intensification (21). Deng et al. subsequently examined the application of TORS in head and neck tumors using a Web of Science Core Collection-based bibliometric approach (22). These studies characterized the broader development of TORS research but did not specifically examine the post-TORS radiotherapy interface. To our knowledge, no previous bibliometric study has focused on the integration of pathological risk assessment, postoperative RT and CRT, radiation dose and target-volume modification, treatment de-intensification, functional preservation, and oncologic outcomes after TORS.

Accordingly, this study performed a bibliometric and knowledge-mapping analysis of research on radiotherapy integration after TORS in head and neck cancer from 2010 to 2026. We characterized publication and citation trends, leading contributors and collaboration networks, intellectual foundations, and conceptual evolution using co-citation, historiographic, bibliographic-coupling, keyword, thematic, factorial, and exploratory topic-modeling analyses. Particular attention was given to postoperative treatment selection, adjuvant RT and CRT, treatment de-intensification, HPV-associated OPSCC, functional preservation, and oncologic outcomes, with radiotherapy integration—rather than TORS technology alone—serving as the central organizing focus of the analysis.

## Materials and methods

### Study design and search strategy

This study was conducted as a bibliometric and knowledge-mapping analysis of research on radiotherapy (RT) and adjuvant-treatment integration after transoral robotic surgery (TORS) for head and neck cancer. Bibliometric methods were used to characterize publication trends, citation patterns, major contributors, collaboration networks, intellectual structure, and thematic evolution (23). Records were retrieved from the Web of Science Core Collection (WoSCC), Scopus, and PubMed on 1 August 2026, covering publications from 1 January 2010 to 1 August 2026. Searches were restricted to English-language original articles and reviews and combined three concept domains: TORS or related transoral robot-assisted surgery, malignant head and neck tumors, and RT or postoperative/adjuvant treatment. Database-specific syntax was adapted to the indexing structure of each platform. The complete search strategies are provided in **Supplementary Table 1**. The initial search identified 1,365 records, including 566 from WoSCC, 632 from Scopus, and 167 from PubMed. Because 2026 represented an incomplete calendar year, publication trends and citation- or keyword-burst intervals extending into 2026 were interpreted only through the retrieval date.

### Eligibility and study selection

**Figure 1:**
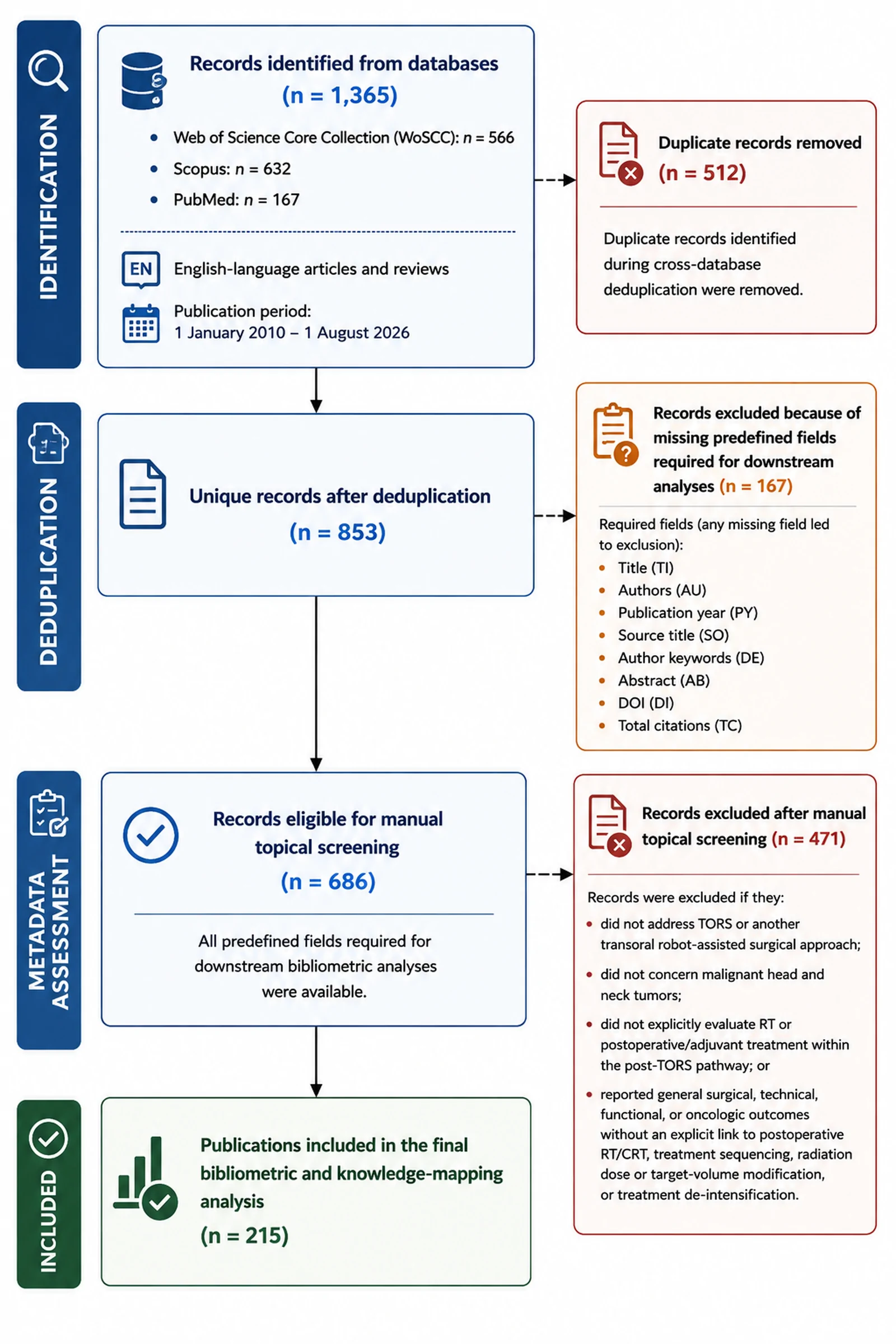
PRISMA-style flow diagram of literature identification, deduplication, metadata-completeness assessment, manual topical screening, and final inclusion.

Studies were eligible if they addressed (1) TORS or another transoral robot-assisted approach, (2) malignant head and neck tumors, and (3) RT or postoperative/adjuvant management as an explicit component of the post-TORS treatment pathway. Relevant concepts included postoperative RT or chemoradiotherapy (CRT), pathologic risk-guided treatment, chemotherapy omission, RT dose or fractionation modification, target-volume modification, omission of irradiation to the resected primary site, treatment sequencing, and postoperative treatment de-intensification. Functional and oncologic outcomes, including swallowing, dysphagia, quality of life, recurrence, and survival, supported inclusion only when explicitly linked to postoperative RT, CRT, radiation modification, or adjuvant-treatment selection. Studies unrelated to malignant head and neck disease, non-oncologic TORS, purely technical or feasibility studies, and publications in which RT or adjuvant therapy was mentioned only incidentally were excluded. After cross-database merging, 512 duplicate records were removed, leaving 853 unique records. Records were subsequently required to contain the predefined fields title (TI), authors (AU), publication year (PY), source title (SO), author keywords (DE), abstract (AB), DOI (DI), and total citation count (TC). Exclusion of 167 records lacking one or more fields required for downstream analyses left 686 records for manual topical screening. Titles were screened first, with abstracts, keywords, and other bibliographic metadata reviewed when eligibility could not be determined from the title alone. A further 471 records were excluded because they did not meet the predefined topical criteria, leaving 215 publications for the final bibliometric and knowledge-mapping analysis. Because individual records could satisfy more than one exclusion criterion, category-specific exclusion counts were not retrospectively assigned. The study-selection process is presented in a PRISMA-style flow diagram (**Figure 1**), adapted for the present bibliometric study (24).

### Data cleaning and bibliometric analysis

Bibliographic metadata were standardized to reduce fragmentation caused by spelling, punctuation, abbreviation, and naming variants. A predefined thesaurus was used to harmonize synonymous terms, including “TORS”/”transoral robotic surgery,” “radiotherapy”/”radiation therapy,” “HPV”/”human papillomavirus,” and “quality-of-life”/”quality of life.” Clinically distinct concepts, including RT, CRT, postoperative RT, dose reduction, target-volume modification, and treatment sequencing, were retained separately. Analyses were performed using bibliometrix/Biblioshiny in R, VOSviewer, and CiteSpace (25–27). Descriptive indicators included annual publication output, citation indicators, source and author productivity, institutional and geographic distribution, and international collaboration. Bradford-type source distributions and Lotka-type author productivity patterns were examined descriptively without assuming formal goodness-of-fit (28, 29). Collaboration networks were evaluated at the author, institution, and country levels. Betweenness centrality, closeness centrality, and PageRank were used to characterize network structure and were not interpreted as direct measures of scientific quality or clinical importance.

### Intellectual and conceptual structure

Global and local citations were analyzed separately. Bibliographic coupling was used to evaluate structural similarity among publications based on shared references, whereas reference co-citation analysis was used to characterize the intellectual foundations of the field (30, 31). Historiographic mapping examined the chronological organization of locally cited publications, and burst analysis identified periods of concentrated citation activity. Keyword analyses included term frequency, co-occurrence, temporal trends, bursts, thematic evolution, and factorial mapping. Thematic evolution was examined across 2010–2021 and 2022–2026. Because the latter interval contained an incomplete 2026 publication year, it was interpreted as representing the literature available through the retrieval date.

### Exploratory publication life-cycle modeling

Cumulative publication output was fitted using a three-parameter logistic model:


$$\text{N}\left(\text{t}\right)=\text{K}/\left\{1\;+\;\exp\left[-\text{r}\left(\text{t}\;-\;{\text{t}}_{\text{m}}\right)\right]\right\}$$

where K represents the estimated asymptotic publication level, r the growth parameter, and t_m_ the inflection point. Model performance was assessed using R², RMSE, AIC, and BIC. Model-derived projections were treated as exploratory descriptions rather than deterministic forecasts.

### Exploratory LDA topic modeling

Latent Dirichlet allocation (LDA) was performed as an exploratory analysis of latent thematic heterogeneity (32, 33). Grid-search optimization evaluated 60 parameter combinations, including K = 5, 10, 15, 20, and 25; α = 0.01, 0.05, and 0.1; and δ = 0.005, 0.01, 0.05, and 0.1. Candidate models were assessed using perplexity, UMass coherence, and bootstrap stability. The selected solution used K = 5, α = 0.01, and δ = 0.1.Because topic-defining terms remained semantically heterogeneous and model stability was limited, LDA findings were interpreted as an exploratory representation of latent thematic structure rather than as definitive research domains.

### Software and reproducibility

Bibliographic data processing and descriptive analyses were conducted using R version 4.6.1 and bibliometrix version 5.4.1, including the Biblioshiny interface. Network construction and visualization were performed using VOSviewer version 1.6.21. Temporal slicing, co-citation visualization, and burst detection were conducted using CiteSpace version 7.0.0 Advanced. All analyses were based on the same finalized and independently verified corpus. The database queries, terminology-harmonization rules, network thresholds, counting and normalization methods, clustering parameters, pruning procedures, processed bibliographic data, screening decisions, and analytical scripts are available from the corresponding author upon reasonable request.

## Results

### General characteristics, temporal evolution, and knowledge linkages

**Figure 2:**
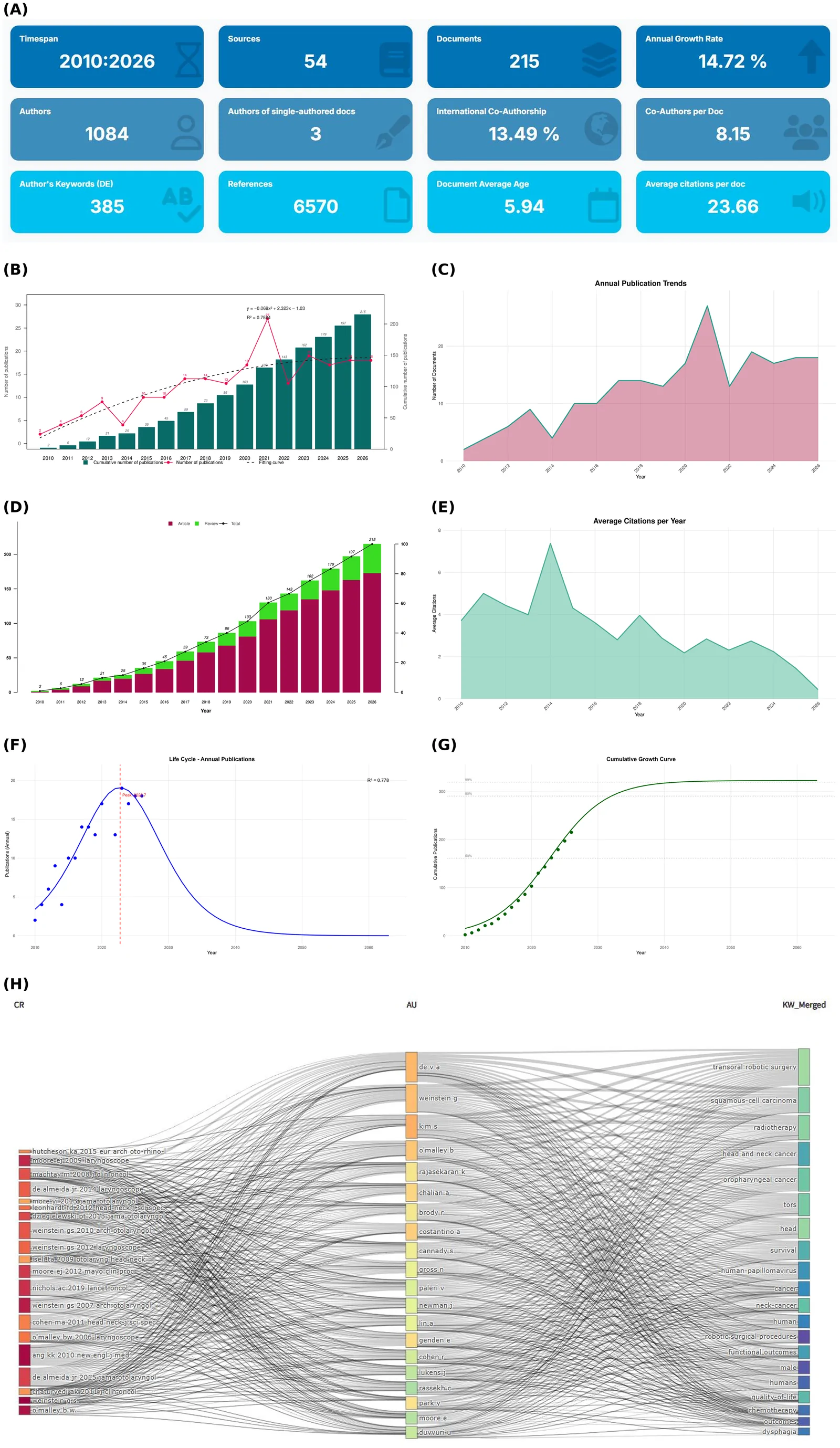
General characteristics, temporal evolution, citation dynamics, life-cycle modeling, and knowledge linkages of research on transoral robotic surgery in head and neck cancer. **(A)** Summary of the main bibliometric characteristics of the included literature. **(B)** Annual and cumulative publication output from 2010 to 2026, together with the fitted temporal trend. **(C)** Annual scientific production showing year-to-year changes in publication activity. **(D)** Temporal distribution of article- and review-type publications and cumulative output. **(E)** Average citations per year across publication cohorts, illustrating the temporal distribution of citation impact. **(F)** Life-cycle model of annual publication output, showing the fitted publication trajectory and model-estimated peak. **(G)** Cumulative logistic growth curve with model-defined 50%, 90%, and 99% saturation thresholds. **(H)** Three-field relationship diagram linking cited references (CR), contributing authors (AU), and merged keywords (KW_Merged), illustrating the connections between the intellectual foundations, major investigators, and principal research topics of the field.

A total of 215 publications published between 2010 and 2026 were included in the final bibliometric analysis, representing 54 sources. The corpus showed an annual growth rate of 14.72%, a mean document age of 5.94 years, and an average of 23.66 citations per publication, with 6,570 cited references, 831 Keywords Plus, and 385 author keywords. A total of 1,084 authors contributed to the literature, with an average of 8.15 co-authors per document; only three publications (1.40%) were single-authored, while international co-authorship accounted for 13.49% of the corpus. These principal bibliometric characteristics are summarized in **Figure 2A**. Article-type publications predominated, comprising 173 records (80.47%), whereas 42 records (19.53%) were classified as reviews (**Figure 2D**). Annual scientific production showed an overall upward trajectory with year-to-year fluctuations, increasing from two publications in 2010 to an empirical maximum of 27 in 2021 (**Figures 2B, C**). Overall, 129 publications (60.0% of the corpus) appeared during 2020–2026. Following a temporary decline to 13 publications in 2022, annual output remained relatively stable at 17–19 publications during 2023–2026. Because 2026 was an incomplete publication year at the time of data retrieval, the 18 publications indexed for that year should be interpreted only through the retrieval date. Citation indicators showed a clear citation-window effect. The 2014 publication cohort had the highest mean total citations per article (95.75) and the highest time-adjusted citation rate (7.37 citations per article per year), followed by the 2011 cohort (80.00 and 5.00, respectively). In contrast, the lower citation rates observed among more recent publication cohorts coincided with substantially shorter citable periods (**Figure 2E**). Exploratory logistic life-cycle modeling yielded a model-estimated asymptote (K) of approximately 323 publications and an inflection point at approximately 2022.7, with a model-defined 10%–90% cumulative-growth interval of approximately 18.6 years. Model performance was characterized by R² = 0.778, RMSE = 2.98, AIC = 43.1, and BIC = 45.6. By 2026, the observed cumulative output of 215 publications corresponded to 66.6% of the estimated asymptote, placing the corpus within the model-defined 50%–90% cumulative-growth interval rather than indicating a defined state of scientific maturity (**Figures 2F, G**). The fitted annual trajectory reached a maximum increment of approximately 19 publications around 2023, whereas the empirical annual maximum was 27 publications in 2021, indicating that the smooth logistic model did not fully reproduce short-term fluctuations in publication activity (**Figure 2F**). Under the fitted model, cumulative output was projected to reach approximately 283 publications by 2031 and 309 by 2036; these estimates were treated as exploratory model-derived projections rather than deterministic forecasts. The three-field analysis showed recurrent relationships among frequently cited references, contributing authors, and merged keywords (**Figure 2H**). Weinstein GS and O’Malley BW were among the prominent authors represented in these linkages, while frequently represented cited references included works by de Almeida, Moore, Nichols, Weinstein, O’Malley, and Ang. These reference–author relationships were connected to dominant terms including transoral robotic surgery, squamous-cell carcinoma, radiotherapy, head and neck cancer, oropharyngeal cancer, and TORS, illustrating the intersection of procedural, oncologic, and radiotherapy-related components within the literature.

### Source productivity, impact, Bradford distribution, and temporal evolution

**Figure 3:**
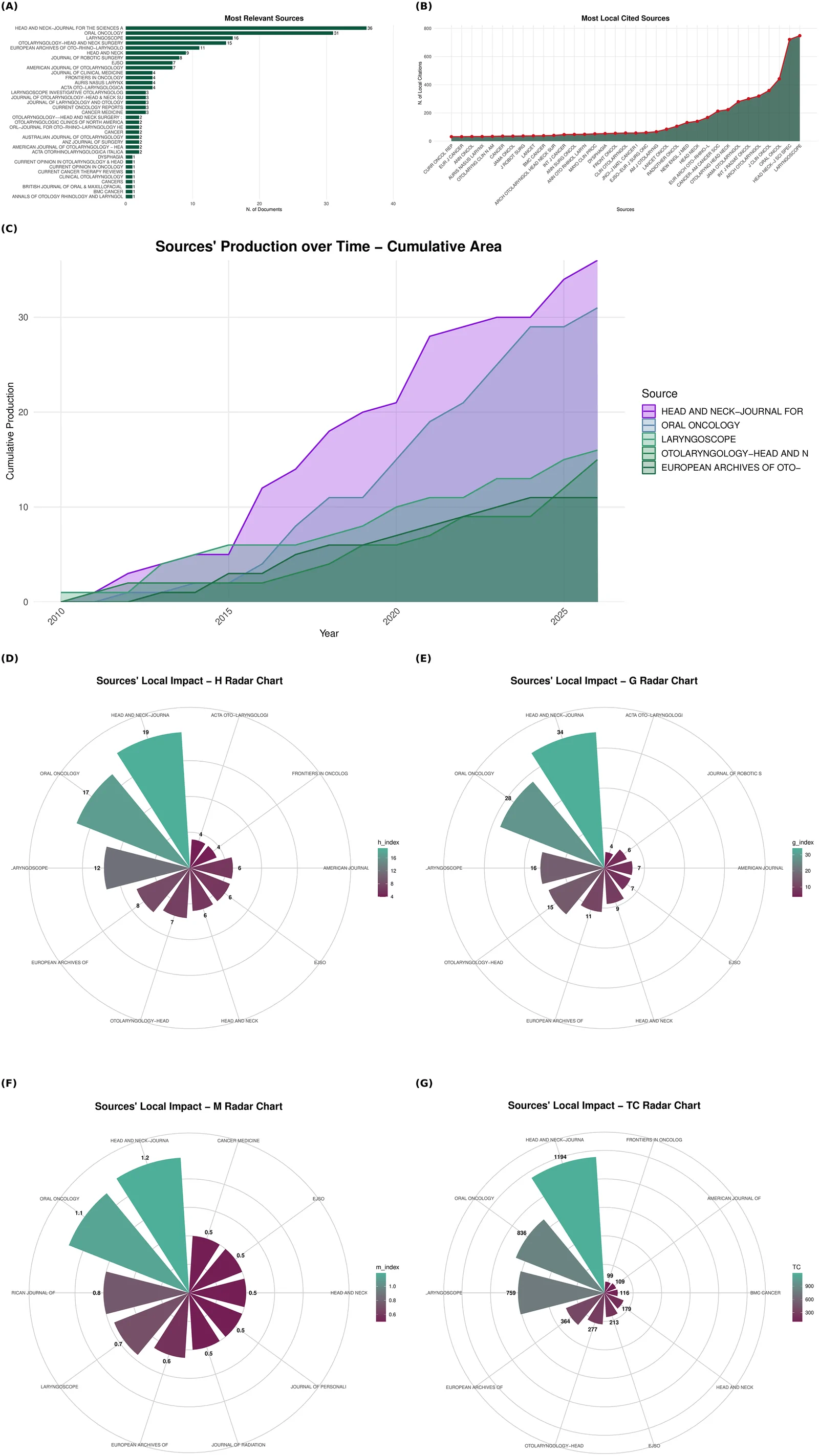
Source productivity, citation impact, and temporal evolution of the literature on transoral robotic surgery in head and neck cancer. **(A)** Most relevant sources ranked by the number of publications. **(B)** Most locally cited sources ranked by local citation frequency within the analyzed corpus. **(C)** Cumulative publication trajectories of the five most productive sources from 2010 to 2026. **(D–G)** Source-level local impact assessed using the h-index **(D)**, g-index **(E)**, m-index **(F)**, and total citations (TC) **(G)**, respectively. Together, these panels characterize the concentration of scientific output, citation influence, and temporal development of the principal journals contributing to the field. Publications indexed in 2026 represent an incomplete publication year at the time of data retrieval.

Source-level analysis showed that publication output was concentrated within a relatively small group of specialist journals. Head and Neck–Journal for the Sciences and Specialties of the Head and Neck was the most productive source, with 36 publications, followed by Oral Oncology (31), Laryngoscope (16), Otolaryngology–Head and Neck Surgery (15), and European Archives of Oto-Rhino-Laryngology (11). Together, these five sources accounted for 109 of the 215 publications (50.7%) (**Figure 3A**). Bradford analysis identified a compact core of productive journals: Zone 1 comprised Head and Neck–Journal for the Sciences and Specialties of the Head and Neck, Oral Oncology, and Laryngoscope, which together contributed 83 publications (38.6%); Zone 2 comprised eight additional sources contributing 69 publications (32.1%); and the remaining 43 sources constituted Zone 3, with 63 publications (29.3%).Source-impact indicators showed a similar concentration among the leading journals (**Figures 3D–G**). Head and Neck–Journal for the Sciences and Specialties of the Head and Neck had the highest h-index (19), g-index (34), m-index (1.188), and total citations (TC = 1,194), followed by Oral Oncology (h = 17, g = 28, m = 1.133, TC = 836) and Laryngoscope (h = 12, g = 16, m = 0.706, TC = 759). Thus, the most productive sources also occupied leading positions across several local impact indicators. The local citation distribution extended beyond the most productive publication sources (**Figure 3B**). Laryngoscope was the most frequently locally cited source, with 748 citations, followed by Head and Neck–Journal for the Sciences and Specialties of the Head and Neck (721) and Oral Oncology (442). Journal of Clinical Oncology (359), Archives of Otolaryngology–Head & Neck Surgery (320), International Journal of Radiation Oncology, Biology, Physics (301), JAMA Otolaryngology–Head & Neck Surgery (280), and Otolaryngology–Head and Neck Surgery (224) were also prominent within the local citation base. Radiotherapy and Oncology and Lancet Oncology were additionally represented among the frequently cited sources, indicating that the cited literature extended beyond surgical and otolaryngology journals to radiation-oncology and broader oncologic sources. Cumulative source-production trajectories showed different temporal patterns among the leading journals (**Figure 3C**). Head and Neck–Journal for the Sciences and Specialties of the Head and Neck maintained the highest cumulative output, increasing from one publication in 2011 to 36 by 2026. Oral Oncology showed a steeper increase after the mid-2010s, rising from four cumulative publications in 2016 to 19 in 2021 and 31 by 2026. Laryngoscope followed a more gradual trajectory and reached 16 publications by 2026. Otolaryngology–Head and Neck Surgery increased from nine cumulative publications in 2024 to 15 by 2026, whereas European Archives of Oto-Rhino-Laryngology reached 11 publications by 2024 and remained unchanged through the retrieval date in 2026.

### Author productivity and citation impact

**Figure 4:**
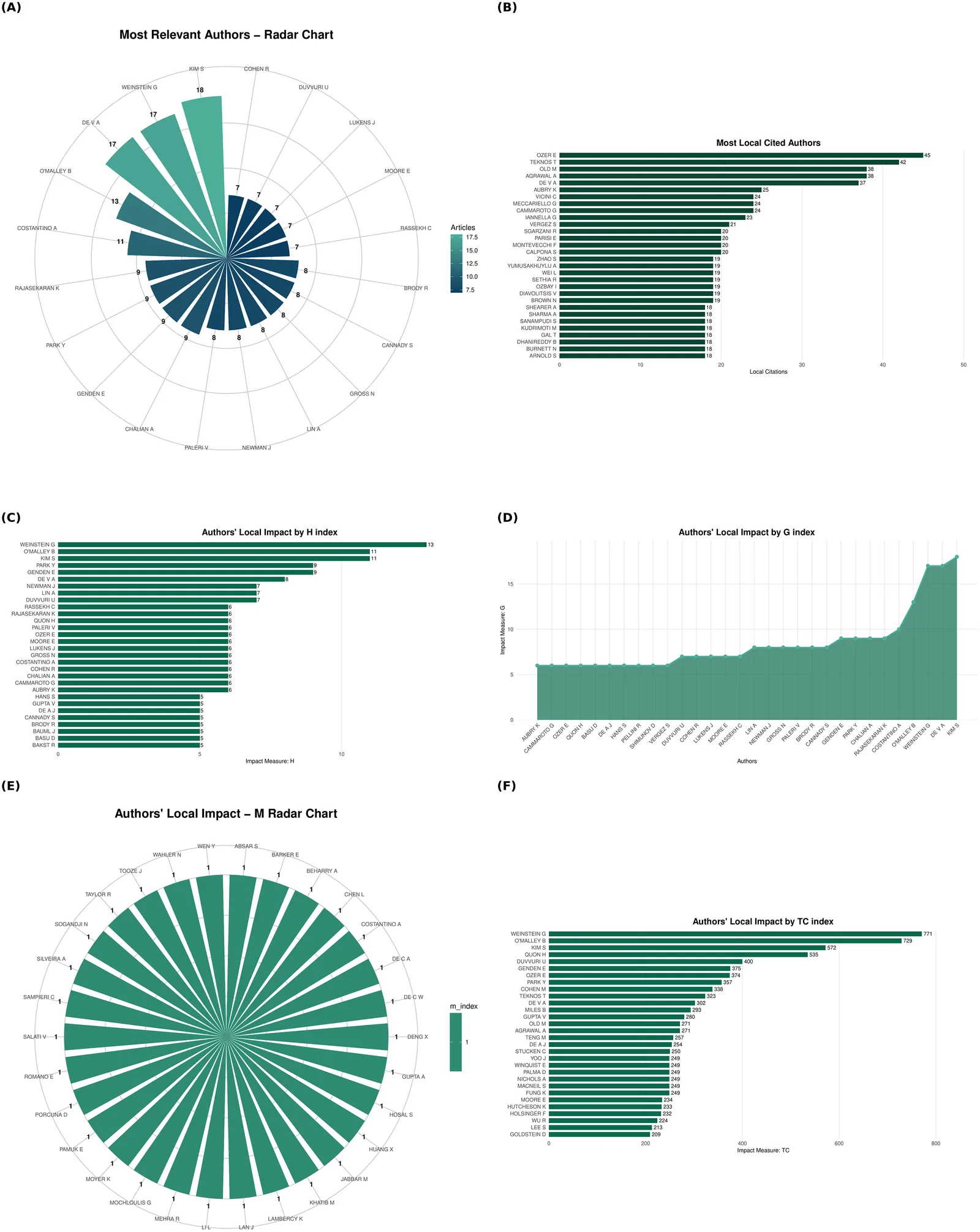
Author productivity, local citation visibility, and author-level impact in the literature on transoral robotic surgery in head and neck cancer. **(A)** Most relevant authors ranked by publication output. **(B)** Most locally cited authors ranked by citation frequency within the analyzed corpus. **(C–F)** Author-level local impact assessed using the h-index **(C)**, g-index **(D)**, m-index **(E)**, and total citations (TC) **(F)**, respectively.

Author-level analyses showed a markedly skewed productivity structure, with a relatively small group of recurrent contributors accounting for a substantial share of publications. Kim S was the most productive author, with 18 publications, followed by Weinstein G and De V A with 17 publications each, O’Malley B with 13, and Costantino A with 11 (**Figure 4A**). Fractionalized publication counts showed a similar pattern, with Kim S ranking first (2.98), followed by Weinstein G (2.11) and De V A (2.01).Local citation rankings differed from publication productivity (**Figure 4B**). Ozer E had the highest number of local citations (45), followed by Teknos T (42), Old M and Agrawal A (38 each), and De V A (37). Thus, De V A combined high publication output with substantial citation visibility within the analyzed corpus. Temporal productivity patterns differed among the leading contributors. Weinstein G and O’Malley B were active from the beginning of the study period. Weinstein G’s publications from 2011 and 2012 accumulated 208 and 201 citations, respectively, and the author contributed five publications in 2021, whereas O’Malley B recorded 201 citations across two publications in 2012 and contributed four publications in 2021. Kim S maintained sustained activity from 2012 to 2025 and published four papers in 2023. De V A showed more concentrated recent productivity, contributing seven publications in 2023—the highest single-year output among the authors evaluated in the temporal analysis—with a TCpY of 16.75. Costantino A likewise contributed six publications in 2023 and three in 2025.Author productivity was strongly right-skewed and broadly resembled a Lotka-type distribution. Among the 1,084 authors, 790 (72.9%) contributed a single publication, 158 (14.6%) contributed two, and 61 (5.6%) contributed three, whereas only two authors contributed 17 publications and one contributed 18. Because the observed proportion of single-publication authors exceeded the theoretical Lotka expectation of 64.1%, strict conformity to Lotka’s law was not inferred in the absence of formal goodness-of-fit testing. Citation-based author-impact indicators further distinguished cumulative and publication-weighted influence (**Figures 4C, D, F**). Weinstein G ranked first by h-index (13) and total citations (TC = 771), while Kim S had the highest g-index (18), together with an h-index of 11, 18 publications, and 572 total citations. O’Malley B also showed substantial cumulative impact, with an h-index of 11 and 729 total citations. High citation accumulation was also observed among authors with smaller publication portfolios, including Quon H (TC = 535; six publications), Duvvuri U (TC = 400; seven publications), and Ozer E (TC = 374; six publications) (**Figure 4F**). The m-index provided a time-normalized complement to these cumulative measures. Costantino A had the highest m-index (1.000), followed by Lukens J and Rajasekaran K (0.857 each) and Basu D and Bauml J (0.833 each). These values indicate comparatively high h-index accumulation relative to the duration of each author’s publication activity. Because the current visualization rounds the displayed m-index values substantially, the exact rankings were derived from the underlying bibliometric output rather than from the plotted labels in **Figure 4E**.

### Institutional, geographic, and funding landscapes

**Figure 5:**
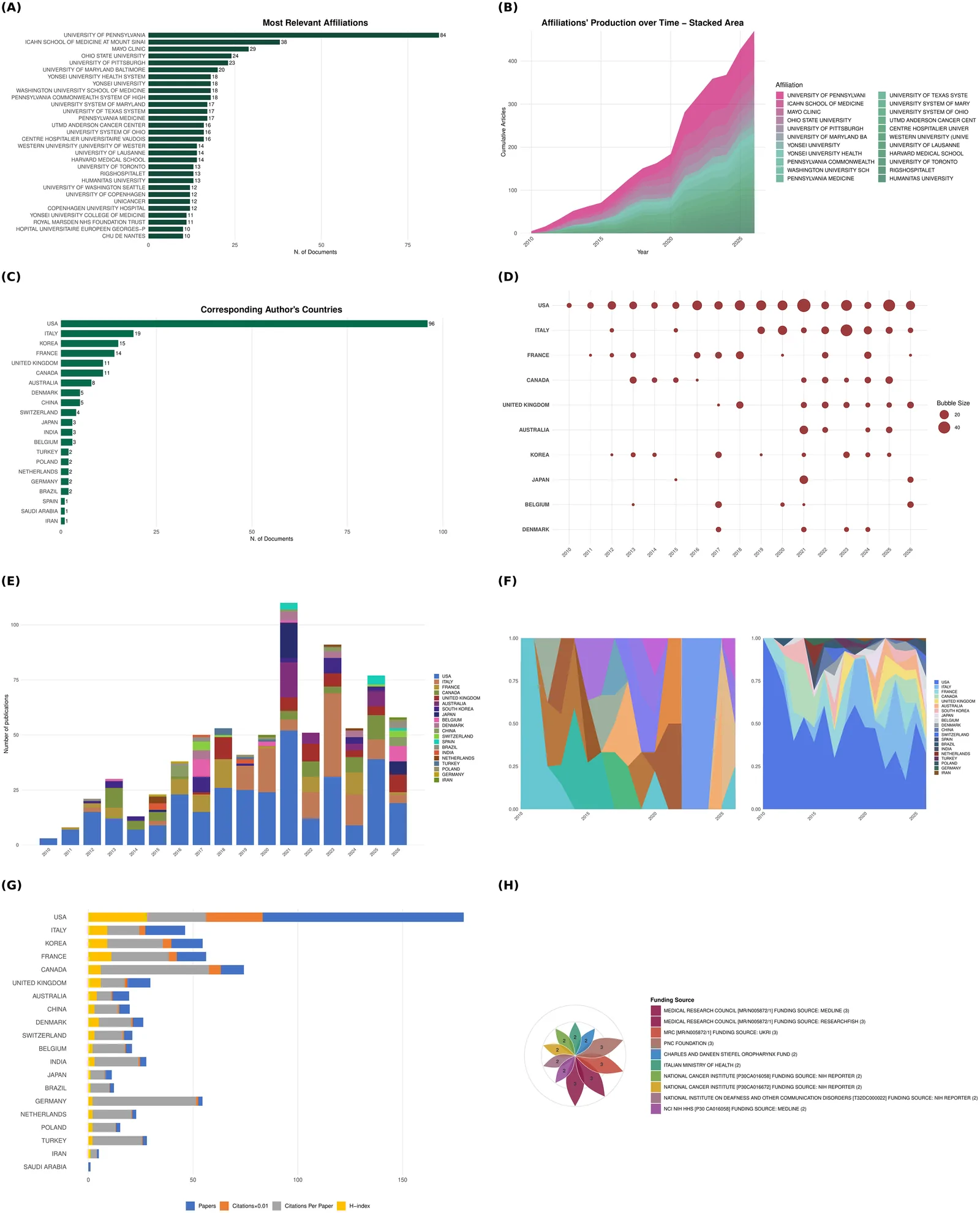
Institutional productivity, geographic distribution, temporal evolution, citation impact, and funding landscape of research on transoral robotic surgery in head and neck cancer. **(A)** Most relevant affiliations ranked by the number of associated publications. **(B)** Cumulative publication trajectories of the leading affiliations over the study period. **(C)** Corresponding-author countries ranked by number of publications. **(D)** Temporal distribution of country-level scientific production, with bubble size representing publication-related frequency in each year. **(E)** Annual country-level scientific production shown as stacked publication counts. **(F)** Temporal changes in the relative contribution of participating countries, displayed as proportional stream plots. **(G)** Country-level citation and impact profile integrating publication count, total citations (scaled by 0.01 for visualization), citations per article, and h-index. **(H)** Major indexed funding sources displayed as a petal plot, with petal values indicating recorded funding-source frequencies.

Institutional and geographic analyses showed a marked concentration of research activity within a relatively limited number of academic centers and countries, together with heterogeneous temporal, collaborative, and citation profiles. Based on indexed affiliation data, the University of Pennsylvania was associated with the largest number of publications (84), followed by the Icahn School of Medicine at Mount Sinai (38), Mayo Clinic (29), Ohio State University (24), and the University of Pittsburgh (23). Other frequently represented affiliations included the University of Maryland Baltimore (20), Washington University School of Medicine (18), Yonsei University (18), UT MD Anderson Cancer Center (16), and Harvard Medical School (14) (**Figure 5A**). Temporal affiliation analysis showed that the University of Pennsylvania maintained the largest cumulative contribution among the institutions included in the analysis, while the overall institutional literature expanded substantially after 2020 (**Figure 5B**). Because individual publications could contain multiple institutional affiliations, these affiliation-linked counts were not mutually exclusive. Corresponding-author country information was available for 210 of the 215 publications. The United States accounted for the largest number of corresponding-author publications (96; 44.7% of the total corpus), followed by Italy (19; 8.8%), South Korea (15; 7.0%), France (14; 6.5%), and Canada and the United Kingdom (11 each; 5.1%) (**Figure 5C**). International collaboration varied substantially across these countries. Multiple-country publications represented 8.3% of US corresponding-author publications, compared with 53.3% for South Korea, 36.4% for Canada, 18.2% for the United Kingdom, and 15.8% for Italy. Country scientific production based on author-affiliation occurrences showed a similar geographic hierarchy but represented a different, non-mutually-exclusive measure of contribution. The United States ranked first with 328 affiliation occurrences, followed by Italy (117), France (55), Canada (50), the United Kingdom (46), Australia (31), South Korea (28), and Japan (23). Temporal production patterns showed sustained US predominance throughout the study period, whereas Italian research activity increased more prominently in the later years; France and Canada showed more gradual expansion, while growth in the United Kingdom was concentrated mainly after 2017 (**Figures 5D, E**). Because these values were based on author-affiliation occurrences, they should not be interpreted as counts of unique publications by country. Citation impact based on corresponding-author country was likewise concentrated in the United States, which accumulated 2,706 total citations, followed by Canada (567), South Korea (399), France (383), and Italy (290) (**Figure 5G**). Rankings based on average citations per article differed from those based on cumulative citation counts. Among the major contributing countries, Canada had the highest average citation rate (51.5 citations per article), followed by the United States (28.2), France (27.4), and South Korea (26.6). Germany also showed a comparatively high average of 49.5 citations per article, although this estimate was based on only two publications and should therefore be interpreted cautiously. Funding information was heterogeneous and included governmental, institutional, and philanthropic support. Frequently indexed sources included the UK Medical Research Council, the PNC Foundation, the Charles and Daneen Stiefel Oropharynx Fund, the Italian Ministry of Health, and several US National Institutes of Health and National Cancer Institute programs (**Figure 5H**). Identical grant identifiers, notably MR/N005872/1 and P30CA016058, appeared under multiple database-specific funding labels. Funding frequencies were therefore interpreted as indexed funding occurrences rather than counts of unique grants or awards.

### Citation impact, intellectual foundations, and citation bursts

**Figure 6:**
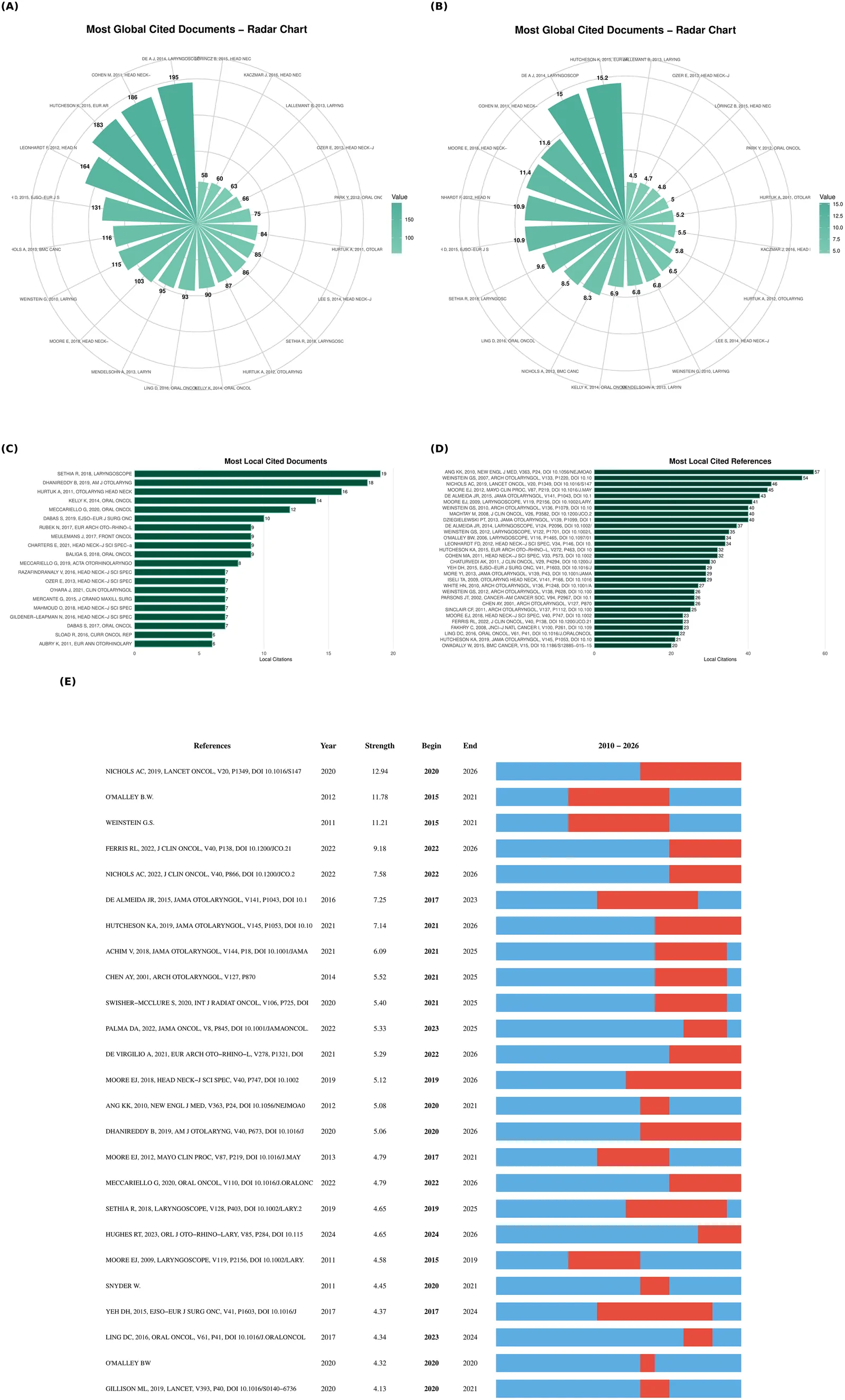
Document-level citation impact, locally cited references, and citation-burst dynamics in research on transoral robotic surgery in head and neck cancer. **(A)** Most globally cited documents ranked by total citations. **(B)** Citation intensity of the leading globally cited documents, expressed as total citations per year. **(C)** Most locally cited documents ranked by the number of citations received from publications within the analyzed corpus. **(D)** Most locally cited references, representing the references most frequently cited by the included publications and thereby characterizing the field’s principal intellectual foundations. **(E)** Citation-burst detection of cited references from 2010 to 2026; burst strength quantifies the intensity of a transient increase in citation activity, and red segments denote the periods during which individual references exhibited citation bursts.

Document- and reference-level citation analyses revealed complementary patterns of cumulative citation impact, citation rates, within-corpus citation concentration, foundational reference use, and temporally concentrated citation activity. Among the included documents, De Almeida JR (2014, Laryngoscope) was the most globally cited publication, with 195 total citations, followed by Cohen M (2011, Head & Neck) with 186 and Hutcheson K (2015, European Archives of Oto-Rhino-Laryngology) with 183 (**Figure 6A**). Annualized citation rates produced a somewhat different ranking: Hutcheson K had the highest rate among the leading documents (15.25 citations per year), followed by De Almeida JR (15.00), while several more recent publications also showed comparatively high citation rates despite lower cumulative citation counts (**Figure 6B**). Additional normalized citation analysis identified Hutcheson K as having the highest normalized global citation score (3.54), with relatively high normalized scores also observed for more recent publications by Lechien J (2020), Moore E (2018), and Miles B (2021).Local citation analysis identified a different hierarchy within the study corpus (**Figure 6C**). Sethia R (2018, Laryngoscope) received the highest number of local citations (19), followed by Dhanireddy B (2019) with 18 and Hurtuk A (2011) with 16. Meccariello G (2020) had the highest normalized local citation score (10.20). The ratio of local to global citations further distinguished documents according to the relative concentration of their impact within the analyzed literature, reaching 50.0% for both Dhanireddy B (2019) and O’Hara J (2021) and 44.44% for Costantino A (2023). Thus, high cumulative global citation counts were not necessarily accompanied by a similarly high concentration of citations within the study corpus. At the cited-reference level, Ang KK et al. (2010, New England Journal of Medicine) was the most frequently cited reference within the corpus, with 57 local citations, followed by Weinstein GS et al. (2007) with 54, Nichols AC et al. (2019) with 46, Moore EJ et al. (2012) with 45, and De Almeida JR et al. (2015) with 43 (**Figure 6D**). Frequently cited references included both TORS-related studies by Weinstein GS, Moore EJ, De Almeida JR, and O’Malley BW and broader head-and-neck oncology studies by Ang KK, Machtay M, Chaturvedi AK, Fakhry C, Parsons JT, and Chen AY. The cited-reference structure therefore reflected the intersection of surgical TORS research with evidence concerning HPV-associated prognosis, oncologic risk, and multimodality treatment.Citation-burst analysis demonstrated temporal changes in concentrated reference use (**Figure 6E**). Nichols AC (2019, Lancet Oncology) exhibited the strongest burst (strength = 12.94), beginning in 2020 and remaining active through the 2026 retrieval date. Strong earlier bursts were also identified for reference records associated with O’Malley BW (strength = 11.78; 2015–2021) and Weinstein GS (11.21; 2015–2021). More recent references with bursts extending through the retrieval date included Ferris RL (2022, Journal of Clinical Oncology; strength = 9.18; 2022–2026), Nichols AC (2022, Journal of Clinical Oncology; 7.58; 2022–2026), Hutcheson KA (2019, JAMA Otolaryngology–Head & Neck Surgery; 7.14; 2021–2026), and De Virgilio A (2021, European Archives of Oto-Rhino-Laryngology; 5.29; 2022–2026). Because 2026 was incomplete at the time of retrieval, bursts ending in 2026 indicate sustained citation activity through 1 August 2026, rather than activity across the complete calendar year.

### High-frequency terms, trend topics, and keyword bursts

**Figure 7:**
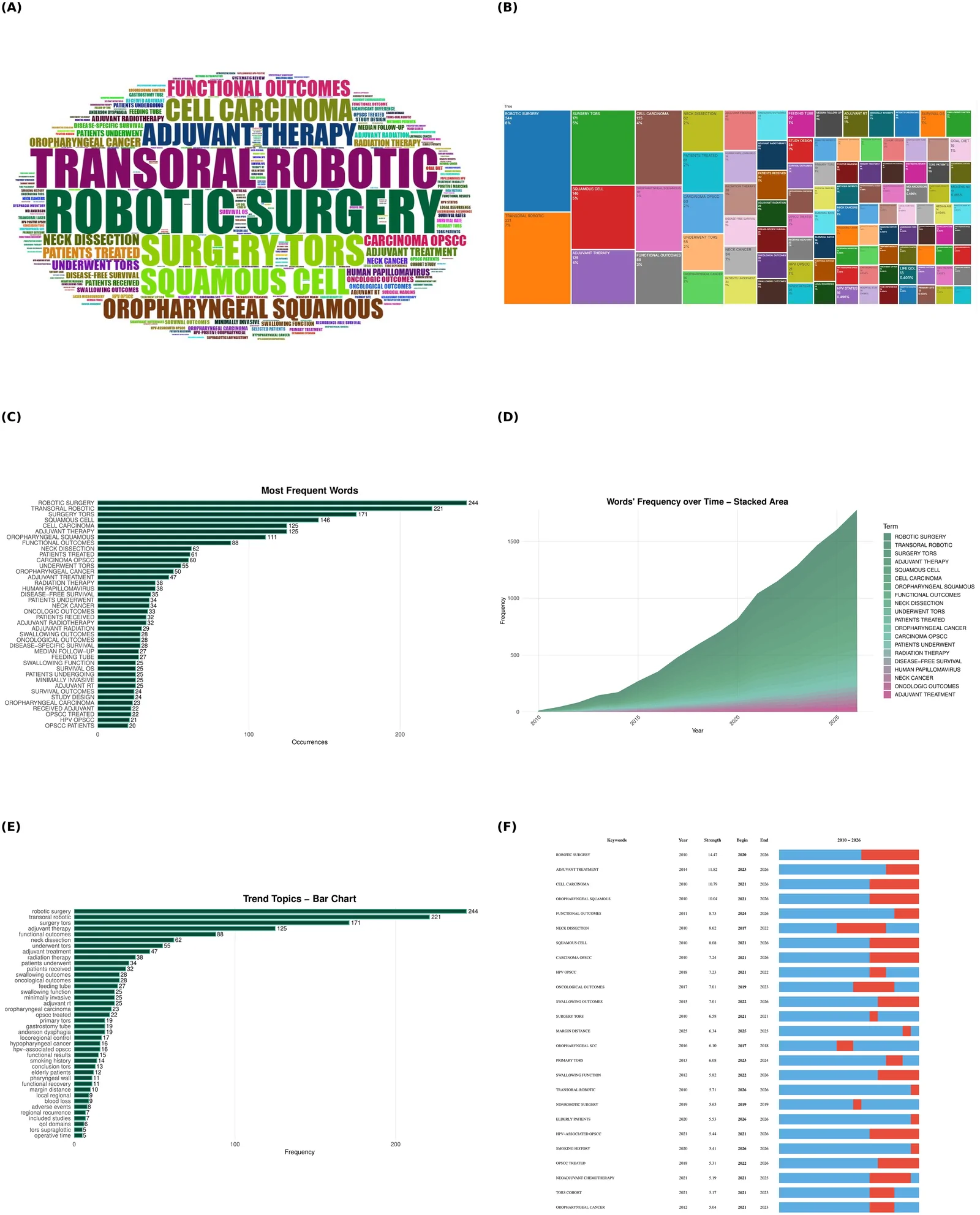
Term-frequency patterns, temporal evolution, trend topics, and keyword bursts in research on transoral robotic surgery in head and neck cancer. **(A)** Word cloud of frequently occurring terms, with word size proportional to occurrence frequency. **(B)** Treemap of high-frequency terms, in which rectangle size represents relative term frequency. **(C)** Most frequent terms ranked by number of occurrences. **(D)** Cumulative temporal trajectories of the leading terms from 2010 to 2026, illustrating their accumulation across the study period. **(E)** Trend-topic terms ranked by frequency, highlighting recurrent procedure-, treatment-, disease-, and outcome-related concepts. **(F)** Keyword citation-burst analysis from 2010 to 2026; burst strength reflects the intensity of a transient increase in term activity, and red segments indicate the corresponding burst periods.

Term-frequency, temporal-trend, and burst analyses showed that the literature was dominated by TORS- and OPSCC-related terminology, with increasing representation of adjuvant treatment and functional outcomes. Across the corpus, the most frequent terms were “robotic surgery” (244 occurrences), “transoral robotic” (221), and “surgery TORS” (171), followed by “squamous cell” (146), “cell carcinoma” (125), and “oropharyngeal squamous” (111) (**Figures 7A–C**). Postoperative-management terminology was also prominent, including “adjuvant therapy” (125), “adjuvant treatment” (47), “radiation therapy” (38), and “adjuvant radiotherapy” (32). Functional, surgical, and outcome-related terms included “functional outcomes” (88), “neck dissection” (62), “disease-free survival” (35), and “oncologic outcomes” (33) (**Figure 7C**). Temporal frequency trajectories demonstrated continued accumulation of the major procedural, oncologic, adjuvant-treatment, and functional terms across the study period (**Figure 7D**). In particular, TORS-related terminology remained prominent throughout the corpus, while terms related to adjuvant treatment and functional outcomes became increasingly represented in the later years. The trend-topic analysis likewise identified “robotic surgery,” “transoral robotic,” “surgery TORS,” “adjuvant therapy,” “functional outcomes,” “neck dissection,” “adjuvant treatment,” and “radiation therapy” among the most frequently represented thematic terms (**Figure 7E**). Keyword-burst analysis provided clearer temporal discrimination of concentrated attention (**Figure 7F**). “Robotic surgery” exhibited the strongest burst (strength = 14.47; 2020–2026), followed by “adjuvant treatment” (11.82; 2023–2026), “cell carcinoma” (10.79; 2021–2026), “oropharyngeal squamous” (10.04; 2021–2026), and “functional outcomes” (8.73; 2024–2026). “Neck dissection” showed an earlier burst from 2017 to 2022 (strength = 8.62), whereas “swallowing outcomes” (7.01; 2022–2026) and “swallowing function” (5.82; 2022–2026) remained active through the retrieval date. “HPV-associated OPSCC” also showed an ongoing burst from 2021 through 2026 (strength = 5.44). Together, these patterns indicate that recent concentrated attention has involved postoperative treatment, HPV-associated disease, and swallowing-related functional outcomes in addition to the persistent procedural focus on TORS. Because 2026 was incomplete at the time of data retrieval, burst intervals ending in 2026 denote activity observed through 1 August 2026, rather than across the complete calendar year.

### Conceptual structure and thematic evolution

**Figure 8:**
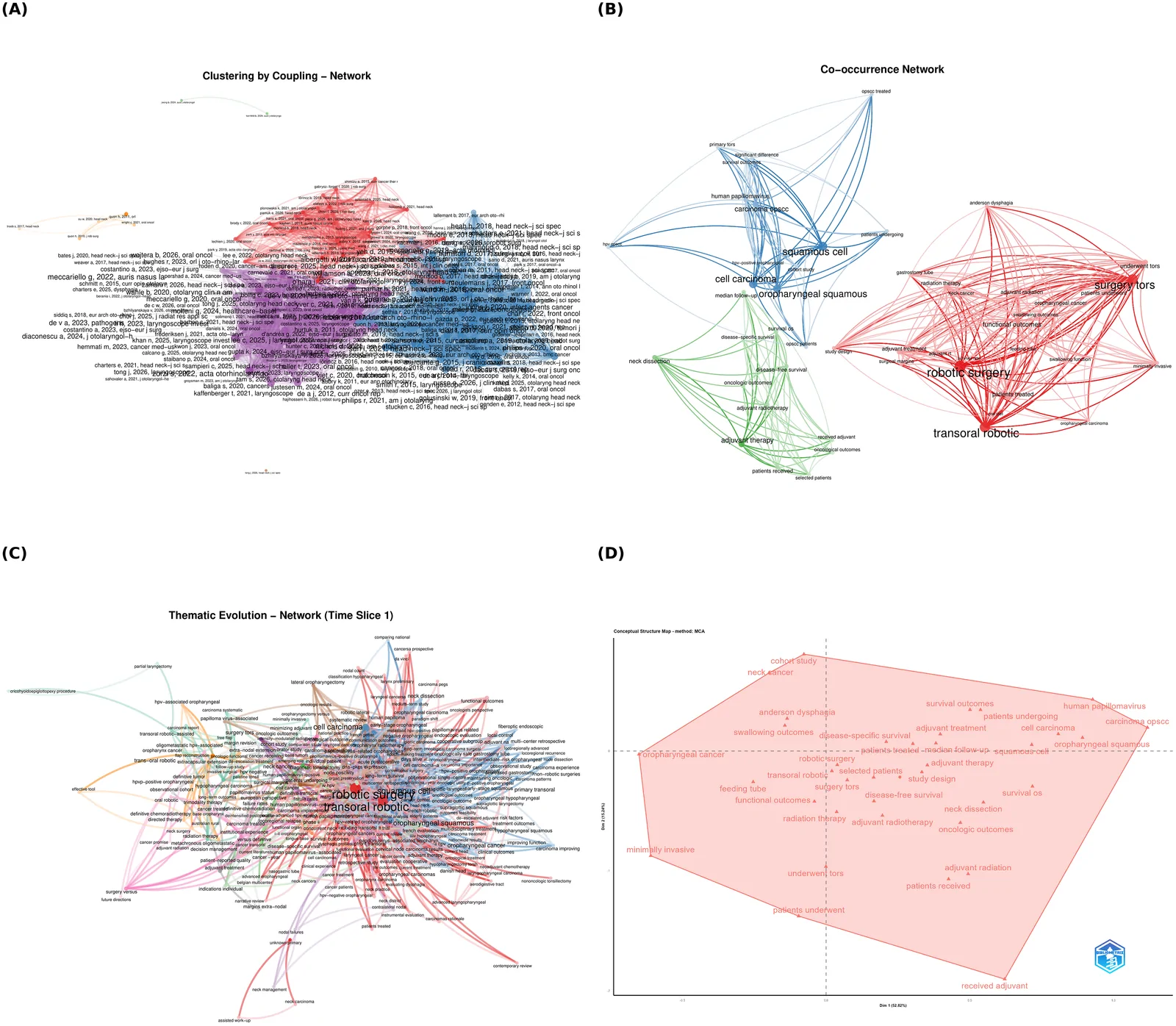
Conceptual structure, thematic clustering, and evolution of research on transoral robotic surgery in head and neck cancer. **(A)** Bibliographic coupling network illustrating structural relationships among publications and the major coupling communities identified from shared reference patterns. **(B)** Keyword co-occurrence network showing three interconnected conceptual domains centered on TORS-related procedures, OPSCC/HPV-related disease characteristics, and adjuvant treatment with oncologic and functional outcomes. **(C)** Thematic evolution network for the first time slice, depicting relationships among procedure-, disease-, treatment-, and outcome-related terms within the evolving knowledge structure. **(D)** Conceptual structure map generated using multiple correspondence analysis (MCA), displaying the relative positions of major terms along the first two dimensions (Dim 1, 52.82%; Dim 2, 15.24%) and illustrating conceptual proximity rather than discrete thematic separation.

Bibliographic coupling, keyword co-occurrence, thematic evolution, and factorial analyses collectively revealed a concentrated conceptual structure with broader thematic representation in the later period. Bibliographic coupling identified eight structural clusters among 212 units included in the coupling solution, although three large communities accounted for 202 units (95.3%). Cluster 2 was the largest (n = 96) and showed the highest impact metric (1.904; centrality = 0.508), followed by Cluster 4 (n = 74; impact metric = 1.713; centrality = 0.430), whereas Cluster 1 (n = 32) exhibited the highest centrality (0.524). All three major clusters were predominantly labeled by robotic surgery, transoral robotic, and surgery TORS, indicating substantial structural concentration but limited semantic differentiation; the remaining clusters were small and showed less coherent or poorly discriminating labels. Keyword co-occurrence analysis yielded three more semantically interpretable but interconnected communities across 50 nodes: a TORS-centered procedural, functional, and treatment-related cluster; an OPSCC/HPV-related disease cluster; and an adjuvant-treatment and oncologic-outcome cluster. Robotic surgery occupied the most prominent structural position in the network (betweenness = 75.762; PageRank = 0.081), followed by transoral robotic (59.656; 0.073) and surgery TORS (54.550; 0.071), while squamous cell, cell carcinoma, and oropharyngeal squamous were prominent within the disease-related cluster and adjuvant therapy showed the highest structural prominence within the postoperative-treatment cluster. Thematic evolution analysis showed that robotic surgery and squamous cell remained dominant across both time slices, although their combined normalized thematic weight decreased from approximately 91.1% in 2010–2021 to 81.9% in 2022–2026. The later period showed greater representation of oropharyngeal cancer (10.04%), functional outcomes (3.21%), swallowing outcomes (1.61%), and HPV-related oropharyngeal (1.20%), indicating a broadening of thematic representation toward disease-specific and functional-outcome topics without displacement of the core TORS-related themes. Factorial analysis did not reveal additional discrete conceptual separation: although the analyzed terms were distributed across the first two dimensions, all were assigned to a single cluster (**Figure 8**). Disease-specific OPSCC/HPV terminology tended to occupy one region of the factorial space, whereas several treatment-related expressions were positioned elsewhere, but these patterns were interpreted cautiously because the factorial solution did not identify multiple stable communities.

### Co-citation, historiographic, and collaboration networks

**Figure 9:**
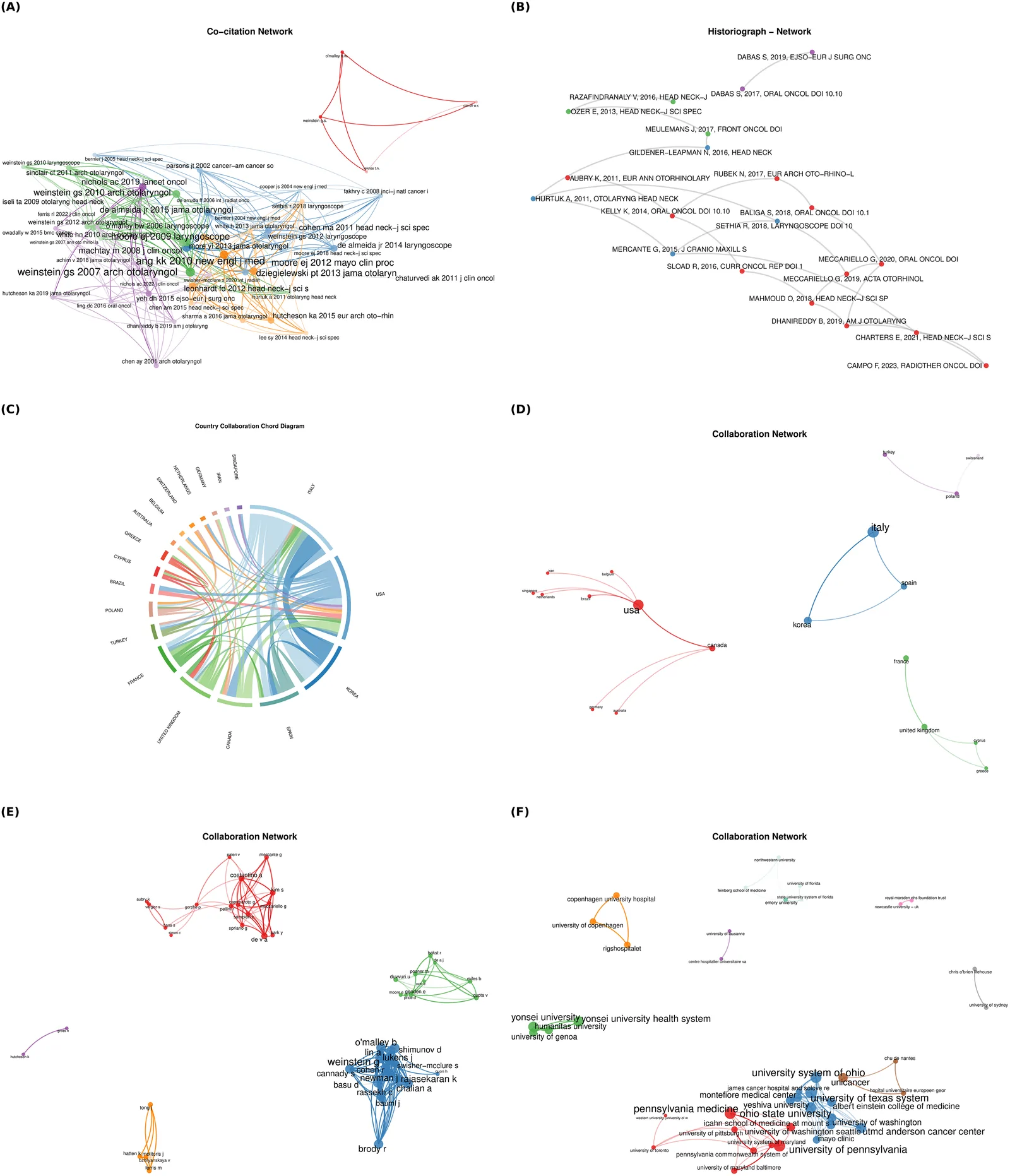
Intellectual foundations, historical development, and collaboration structure of transoral robotic surgery research in head and neck cancer. **(A)** Reference co-citation network showing the major intellectual communities formed by frequently co-cited landmark studies, with node size and network position reflecting their structural prominence within the shared knowledge base. **(B)** Historiographic network depicting the chronological relationships among locally influential publications and the evolution of research from early TORS feasibility and oncologic evaluation toward adjuvant treatment optimization and functional outcomes. **(C)** Country collaboration chord diagram illustrating cross-national collaborative relationships among contributing countries. **(D)** Country collaboration network showing the principal international collaboration clusters and the structural positions of highly connected countries. **(E)** Author collaboration network depicting major coauthorship communities and bridging investigators within the field. **(F)** Institutional collaboration network illustrating interinstitutional research partnerships and the distribution of major affiliation-based collaboration clusters.

Co-citation, historiographic, and collaboration-network analyses revealed a structured intellectual foundation, an evolving clinical research focus, and a collaboration landscape concentrated around a limited number of highly connected countries, authors, and institutions. Reference co-citation analysis identified five clusters; the interpretable communities encompassed foundational TORS research, HPV-associated oropharyngeal squamous cell carcinoma (OPSCC), multimodality treatment, and functional and oncologic outcomes. Ang KK (2010, New England Journal of Medicine) showed the highest betweenness centrality (32.879), whereas Weinstein GS (2007, Archives of Otolaryngology–Head & Neck Surgery) had the highest PageRank (0.037), indicating prominent bridging and embedded positions, respectively, within the co-citation network. Historiographic analysis identified 20 locally cited landmark documents across four clusters and showed a chronological shift from early clinical implementation and oncologic evaluation of TORS toward disease-specific applications, comparisons with chemoradiotherapy, refinement of adjuvant treatment, and increasing attention to swallowing, quality of life, and functional preservation. Sethia R (2018) had the highest local citation score among these documents (LCS = 19), whereas Kelly K (2014) had the highest global citation score (GCS = 90). At the country level, 19 countries were distributed across four collaboration clusters, with the United States occupying the most central position (betweenness = 94.563; PageRank = 0.183); Canada (betweenness = 40.200), Italy (39.536), and the United Kingdom (34.184) also showed substantial bridging roles. Author collaboration analysis identified 49 authors across five clusters, with three major communities accounting for most of the connected structure. Duvvuri U (betweenness = 442.750) and Kim S (391.602) showed the strongest bridging positions, while De V A had the highest PageRank (0.043); Weinstein G, O’Malley B, Genden E, and Costantino A also occupied prominent network positions. Institutional collaboration involved 41 affiliation nodes distributed across 10 clusters. Icahn School of Medicine at Mount Sinai had the highest betweenness centrality (42.996), followed by Unicancer (40.000) and the University of Pennsylvania (32.567), whereas the University of Pennsylvania had the highest PageRank (0.037)(**Figure 9**).

### Exploratory LDA topic modeling

**Figure 10:**
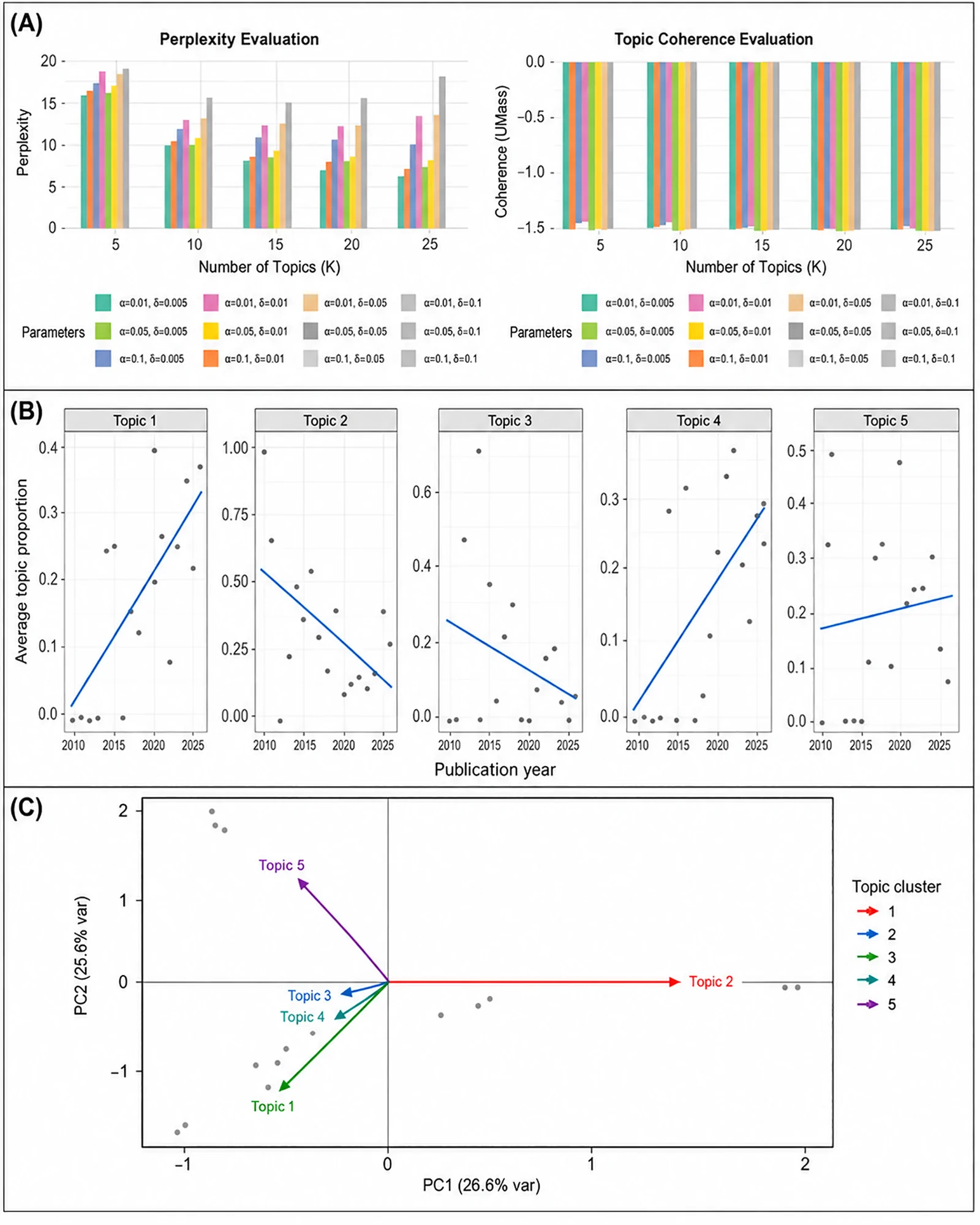
Model optimization, temporal prevalence, and latent-topic structure of the exploratory LDA analysis. **(A)** Grid-search evaluation of candidate LDA models across topic numbers (K = 5, 10, 15, 20, and 25) and combinations of the document–topic prior (α) and topic–term prior (δ), assessed using perplexity and UMass topic coherence. The selected model used K = 5, α = 0.01, and δ = 0.1. **(B)** Temporal variation in the average prevalence of the five latent topics across publication years; gray points represent annual average topic proportions and blue lines indicate fitted linear trends. **(C)** Two-dimensional principal-component representation of the latent-topic space, with PC1 and PC2 explaining 26.6% and 25.6% of the variance, respectively.

Grid-search optimization across 60 parameter combinations selected a five-topic solution (K = 5, α = 0.01, δ = 0.1) according to the predefined model-selection procedure. The model yielded a perplexity of 18.66, a coherence score of −1.4546, and a bootstrap stability of 0.3356. Topic prevalence ranged from 14.35% to 25.92%, with no single topic dominating the corpus. Topic 2 was the most prevalent (25.92%) and included terms related to early-stage disease, neck dissection, functional and oncologic outcomes, and swallowing. Topic 5 (21.53%) was associated with radiation therapy, salvage treatment, upfront surgery, and laryngectomy, whereas Topic 1 (21.20%) included clinical experience, functional evaluation, HPV-associated disease, and transoral surgical management. Topic 4 (17.00%) was characterized by chemoradiation, definitive treatment, HPV-associated oropharyngeal cancer, and adjuvant treatment, while Topic 3 (14.35%) primarily contained HPV-related, recurrence-, risk-, and outcome-related terms. Because several topic-defining terms remained heterogeneous and the model showed limited stability, the LDA solution was interpreted exploratorily as a representation of latent thematic heterogeneity rather than as a set of discrete, definitive research domains (**Figure 10**).

## Discussion

The present analysis shows that research on radiotherapy (RT) integration after transoral robotic surgery (TORS) has moved beyond an early emphasis on procedural feasibility and oncologic validation toward postoperative risk stratification, adjuvant RT or chemoradiotherapy (CRT), treatment de-intensification, and functional preservation. Sixty percent of the included publications appeared between 2020 and 2026, and recent citation and keyword activity increasingly involved adjuvant treatment, HPV-associated oropharyngeal squamous cell carcinoma (OPSCC), and swallowing-related outcomes. The citation structure showed a similar pattern, with foundational TORS studies embedded within a knowledge base that also incorporated evidence on HPV-associated prognosis and postoperative RT/CRT. Compared with previous bibliometric studies addressing the broader TORS literature (21, 22), the present analysis therefore identifies postoperative treatment integration as a distinct organizing problem. This interpretation is consistent with the recent ASCO guideline, which places TORS within a multidisciplinary framework encompassing patient selection, pathological assessment, adjuvant therapy, and functional outcomes (34).

A central feature of this transition is the use of surgical pathology to determine subsequent treatment rather than to justify treatment reduction by default. E3311 established a risk-adapted postoperative framework in which observation, 50-Gy RT, 60-Gy RT, or CRT was assigned according to pathological findings (13, 14). Long-term follow-up showed sustained disease control across the risk-defined arms, although recurrences were observed in each treatment group (14). Observational evidence provides an important counterpoint. Carey et al. reported a 3-year locoregional failure rate of 32% among patients with HPV-associated OPSCC who did not receive recommended postoperative RT after TORS, compared with 4% among those who received it (35). Zebolsky et al. further found pathological extranodal extension (ENE) and/or positive margins in 25% of carefully selected surgical patients without obvious clinical ENE (36). These findings illustrate a fundamental limitation of preoperative de-intensification strategies: clinical selection alone cannot reliably identify all patients who will ultimately avoid postoperative RT or CRT. One of the principal contributions of TORS is therefore the pathological information it provides, which may support treatment reduction in some patients but indicate treatment intensification in others.

Postoperative dose reduction has consequently been investigated in patients for whom adjuvant treatment remains indicated but whose clinical and pathological characteristics permit consideration of a lower-intensity regimen. MC1273 evaluated 30–36 Gy with docetaxel after margin-negative resection in selected patients with HPV-associated OPSCC and reported a 2-year locoregional control rate of 96.2%, together with low late toxicity and limited deterioration in swallowing and quality of life (37). Longer follow-up confirmed favorable functional recovery and a low incidence of clinically important late toxicity (38). These findings provided a rationale for testing substantial postoperative dose reduction prospectively, while also underscoring that such strategies were developed within carefully selected populations rather than as general substitutes for standard adjuvant treatment.

MC1675 extended this approach into a randomized phase III setting. The de-escalated adjuvant regimen of 30–36 Gy with docetaxel was associated with a lower cumulative incidence of grade ≥3 chronic toxicity between 3 and 24 months (3% vs. 11%) and lower cumulative gastrostomy-tube use (2% vs. 8%) than standard postoperative treatment (39). The oncologic findings, however, also defined an important boundary to uniform de-intensification. Progression-free survival was less favorable with de-escalated adjuvant treatment in higher-risk subgroups, particularly in patients with pN2 disease and ENE, indicating that the safety of substantial dose reduction is not uniform across pathological risk groups (39). MC1675 should therefore be interpreted as evidence that a substantially de-intensified postoperative regimen can reduce treatment morbidity in appropriately selected patients, not as support for indiscriminate postoperative dose reduction. This risk-dependent interpretation is consistent with the subsequent analysis by Hidalgo et al., in which 2-year progression-free survival was 93% both among patients treated within the MC1273/MC1675 trials and among selected patients treated subsequently in clinical practice; however, follow-up was shorter and pN2 disease was less frequent in the off-study cohort (40).

Research has also moved beyond dose reduction toward modification of the irradiated volume. AVOID evaluated omission of intentional irradiation to the resected primary-site bed in selected patients with favorable primary-site pathology who continued to receive indicated nodal treatment (17). In a related unknown-primary setting, the FIND trial applied a similar principle to p16-positive cervical squamous cell carcinoma of unknown primary origin, where TORS-assisted localization enabled reduction of pharyngeal and neck RT volumes while maintaining favorable disease control in a phase II study (41). Dosimetric analyses have likewise shown that omission of the resected primary bed can reduce radiation exposure to adjacent organs at risk (42). These studies distinguish two clinically different forms of postoperative de-intensification: reduction of the prescribed dose and reduction of the volume of tissue exposed to radiation. Both approaches may lessen treatment burden, but their appropriateness remains dependent on the pathological and anatomical assessment of residual risk.

The growing representation of swallowing and functional outcomes in the bibliometric analysis is consistent with this evolution in clinical priorities. In E3311, swallowing outcomes worsened after transoral surgery and subsequently improved during follow-up; among intermediate-risk patients, aspiration and high-grade dysphagia were less frequent after 50 Gy than after 60 Gy at 6 months, although these differences were not maintained at 24 months (15). ORATOR similarly demonstrated different toxicity and quality-of-life profiles for primary RT and TORS-based management rather than a consistent functional advantage for either strategy (11, 12). ORATOR2 provides an additional caution: the trial was terminated early after two treatment-related deaths in the surgical arm, and its small sample size limits generalization of the observed survival differences (43). Taken together, these studies argue against using “TORS” or “RT” as proxies for treatment burden. Functional outcome is determined by the complete treatment sequence, including surgery, neck dissection, postoperative RT or CRT, radiation dose and volume, systemic therapy, and subsequent rehabilitation.

This distinction is particularly important in HPV-associated OPSCC, which accounts for much of the contemporary de-intensification literature. The favorable prognosis of HPV-positive disease provides a strong rationale for reducing late morbidity, but HPV status alone does not define a postoperative low-risk population. Smoking history, primary and nodal stage, nodal burden, margin status, ENE, lymphovascular invasion, and perineural invasion remain relevant to postoperative treatment selection (34, 44–46). Reviews of HPV-associated and surgically based de-intensification have accordingly emphasized selective, risk-adapted treatment rather than broadly applied reduction (44–46). The recent prominence of “HPV-associated OPSCC” and “adjuvant treatment” in our analysis should therefore be interpreted as evidence of continued refinement of postoperative selection criteria rather than as evidence that RT omission is becoming standard practice.

Efforts to improve this selection are increasingly quantitative. Costantino et al. used National Cancer Database data from 5,569 patients with HPV-positive OPSCC treated with transoral surgery to develop machine-learning survival models for postoperative risk stratification (47). The resulting risk groups showed different associations between adjuvant treatment and overall survival. However, the retrospective database design and potential for confounding by indication preclude causal inference regarding treatment benefit. These models are therefore better viewed as candidates for prospective validation and trial stratification than as a current basis for omitting RT.

Biomarker-guided treatment selection remains similarly investigational. In the prospective PATH study, postoperative HPV circulating tumor DNA was used to guide deferral of otherwise indicated adjuvant RT. Three of 12 enrolled patients developed radiographic recurrence; these recurrences were not preceded by detectable circulating HPV DNA, and the cohort was closed according to a prespecified stopping rule (48). This negative result is informative because it illustrates the limitations of replacing established pathological risk criteria with a single molecular marker. Circulating tumor DNA and other molecular residual-disease assays may eventually complement postoperative risk assessment, but the available evidence does not support using ctDNA negativity alone to omit otherwise indicated adjuvant treatment.

The geographic distribution of the literature raises a separate issue of generalizability. The United States accounted for 96 corresponding-author publications, and research output was concentrated among a relatively limited group of specialist academic institutions, while international co-authorship represented only 13.49% of the corpus. These bibliometric findings should not be interpreted as measures of clinical case volume, institutional expertise, or treatment quality. They nevertheless indicate that much of the published evidence has emerged from a relatively concentrated academic network. Because TORS-based management requires coordination among surgery, pathology, radiation oncology, functional assessment, and rehabilitation, broader multicenter and international validation will be important when determining whether treatment strategies developed within specialist centers are reproducible across different practice environments.

The conceptual analyses also favor a restrained interpretation of thematic change. Bibliographic coupling identified eight clusters, but three large communities accounted for 95.3% of clustered publications and remained dominated by overlapping TORS-related terminology. Keyword co-occurrence produced more clinically recognizable procedural/functional, HPV/OPSCC, and adjuvant-treatment groupings, whereas factorial analysis did not identify clearly separable conceptual communities. The LDA model likewise produced five latent topics with substantial semantic overlap and limited bootstrap stability. These findings are consistent with the clinical interdependence of surgery, HPV biology, postoperative RT, swallowing, recurrence, and risk stratification rather than with the existence of sharply separated research domains. Citation and keyword bursts extending through 2026 should therefore be interpreted as markers of concentrated attention through the retrieval date rather than as predictions of future research priorities.

The publication life-cycle analysis warrants similar caution. By 2026, the observed corpus corresponded to 66.6% of the logistic model-estimated asymptote, but the fitted maximum annual increment of approximately 19 publications did not reproduce the empirical peak of 27 publications observed in 2021. The model-defined 50%–90% interval is therefore more appropriately regarded as a feature of the fitted cumulative trajectory than as evidence of scientific or clinical maturity. The continued publication of randomized trials, updated clinical guidance, real-world implementation studies, and model- and biomarker-based investigations during 2025–2026 further argues against interpreting the estimated asymptote as a fixed ceiling for future research activity (34, 39, 40, 47, 48).

### Strengths and limitations

A principal strength of this study is its focus on the post-TORS radiotherapy and adjuvant-treatment interface, rather than on the broader TORS literature. This narrower scope allowed pathological risk stratification, postoperative RT and CRT, dose and target-volume modification, functional outcomes, and oncologic outcomes to be examined within a common framework. Integration of WoSCC, Scopus, and PubMed broadened disciplinary coverage, while manual topical screening reduced the inclusion of general robotic-surgery studies without substantive relevance to postoperative treatment. The combination of citation, network, thematic, historiographic, and exploratory modeling approaches provided complementary views of the field.

Several limitations warrant consideration. Restriction to English-language publications indexed in WoSCC, Scopus, and PubMed may have excluded relevant studies from other databases and non-English literature. Requiring predefined bibliographic fields, including abstracts, author keywords, DOI, and citation information, may have introduced metadata-related selection bias. Citation indicators depend on publication age, database coverage, disciplinary citation practices, and self-citation and should not be regarded as measures of methodological quality. Residual ambiguity in author names and institutional affiliations may remain despite normalization. In addition, much of the clinical literature concerns HPV-associated OPSCC treated at specialist centers, limiting extrapolation to HPV-negative disease, other head and neck subsites, salvage TORS, and different practice environments. Finally, 2026 was an incomplete publication year through 1 August. The logistic model was exploratory, and the limited stability of the LDA solution precludes treating its latent topics as definitive or reproducible research domains.

## Conclusion

Research on radiotherapy integration after TORS has shifted from an emphasis on procedural feasibility toward postoperative risk stratification, risk-adapted radiotherapy, treatment de-intensification, and functional preservation. TORS should not itself be equated with treatment de-intensification; whether it reduces overall treatment burden depends on the pathological findings generated by surgery and the postoperative therapy those findings require. The evidence therefore favors viewing surgery and radiotherapy as components of a risk-adapted treatment pathway rather than assuming a simple substitution between the two modalities.

Further progress will require clearer identification of patients in whom postoperative treatment can be safely reduced and those who continue to require standard RT or CRT. Prospective multicenter validation, standardized pathological and radiotherapy criteria, longitudinal functional assessment, and externally validated risk models will be necessary to define the appropriate limits of postoperative dose reduction, target-volume modification, and treatment omission.

## Data Availability

The original contributions presented in the study are included in the article/**Supplementary Material**. Further inquiries can be directed to the corresponding authors.
