# Supplementary material for: Radiotherapy integration after transoral robotic surgery in head and neck cancer: a bibliometric and knowledge-mapping analysis of adjuvant treatment, de-escalation, functional preservation, and survival outcomes
Source: Front Oncol. 2026 Aug 28;16:1901904. doi: 10.3389/fonc.2026.1901904 (PMC13561835; doi:10.3389/fonc.2026.1901904)
Supplement: Supplementary file 1 [file Table1.docx]

**Supplementary Table S1. Complete database-specific search strategies**

| **Database** | **Search field** | **Search strategy** |
| --- | --- | --- |
| Web of Science Core Collection (WoSCC) | Topic (TS) | TS=( ( "transoral robotic surgery" OR TORS OR "transoral robot-assisted surgery" OR "transoral robot assisted surgery" OR "robot-assisted transoral surgery" OR "robot assisted transoral surgery" OR "robotic transoral surgery" OR "transoral robotic resection" OR "transoral robotic microsurgery" ) AND ( radiotherapy OR "radiation therapy" OR "radiation oncology" OR "adjuvant radiotherapy" OR "adjuvant radiation therapy" OR "postoperative radiotherapy" OR "postoperative radiation therapy" OR "post-operative radiotherapy" OR "post-operative radiation therapy" OR "postoperative chemoradiotherapy" OR "postoperative chemoradiation" OR "adjuvant chemoradiotherapy" OR "adjuvant chemoradiation" OR chemoradiotherapy OR chemoradiation OR "concurrent chemoradiotherapy" OR "concurrent chemoradiation" OR "de-escalation" OR deescalation OR "de-intensification" OR deintensification OR "dose reduction" OR "radiation dose reduction" OR "treatment sequencing" OR "treatment sequence*" OR "multimodal treatment" OR "multimodality treatment" ) AND ( "head and neck cancer" OR "head and neck cancers" OR "head and neck tumor*" OR "head and neck tumour*" OR "head and neck neoplasm*" OR "head and neck squamous cell carcinoma" OR HNSCC OR "oropharyngeal cancer" OR "oropharyngeal carcinoma" OR "oropharyngeal squamous cell carcinoma" OR OPSCC OR "HPV-positive oropharyngeal cancer" OR "HPV positive oropharyngeal cancer" OR "human papillomavirus-positive oropharyngeal cancer" OR "tonsil cancer" OR "tonsillar cancer" OR "base of tongue cancer" OR "tongue base cancer" OR "pharyngeal cancer" OR "laryngeal cancer" OR "hypopharyngeal cancer" ) ) |
| Scopus | TITLE-ABS-KEY | TITLE-ABS-KEY( ( "transoral robotic surgery" OR TORS OR "transoral robot-assisted surgery" OR "transoral robot assisted surgery" OR "robot-assisted transoral surgery" OR "robot assisted transoral surgery" OR "robotic transoral surgery" OR "transoral robotic resection" OR "transoral robotic microsurgery" ) AND ( radiotherapy OR "radiation therapy" OR "radiation oncology" OR "adjuvant radiotherapy" OR "adjuvant radiation therapy" OR "postoperative radiotherapy" OR "postoperative radiation therapy" OR "post-operative radiotherapy" OR "post-operative radiation therapy" OR "postoperative chemoradiotherapy" OR "postoperative chemoradiation" OR "adjuvant chemoradiotherapy" OR "adjuvant chemoradiation" OR chemoradiotherapy OR chemoradiation OR "concurrent chemoradiotherapy" OR "concurrent chemoradiation" OR "de-escalation" OR deescalation OR "de-intensification" OR deintensification OR "dose reduction" OR "radiation dose reduction" OR "treatment sequencing" OR "treatment sequence*" OR "multimodal treatment" OR "multimodality treatment" ) AND ( "head and neck cancer" OR "head and neck cancers" OR "head and neck tumor*" OR "head and neck tumour*" OR "head and neck neoplasm*" OR "head and neck squamous cell carcinoma" OR HNSCC OR "oropharyngeal cancer" OR "oropharyngeal carcinoma" OR "oropharyngeal squamous cell carcinoma" OR OPSCC OR "HPV-positive oropharyngeal cancer" OR "HPV positive oropharyngeal cancer" OR "human papillomavirus-positive oropharyngeal cancer" OR "tonsil cancer" OR "tonsillar cancer" OR "base of tongue cancer" OR "tongue base cancer" OR "pharyngeal cancer" OR "laryngeal cancer" OR "hypopharyngeal cancer" ) ) AND PUBYEAR > 2009 AND PUBYEAR < 2027 AND LANGUAGE(english) AND (DOCTYPE(ar) OR DOCTYPE(re)) |
| PubMed | Title/Abstract | ( "transoral robotic surgery"[Title/Abstract] OR "TORS"[Title/Abstract] OR "transoral robot-assisted surgery"[Title/Abstract] OR "transoral robot assisted surgery"[Title/Abstract] OR "robot-assisted transoral surgery"[Title/Abstract] OR "robot assisted transoral surgery"[Title/Abstract] OR "robotic transoral surgery"[Title/Abstract] OR "transoral robotic resection"[Title/Abstract] OR "transoral robotic microsurgery"[Title/Abstract] ) AND ( "radiotherapy"[Title/Abstract] OR "radiation therapy"[Title/Abstract] OR "radiation oncology"[Title/Abstract] OR "adjuvant radiotherapy"[Title/Abstract] OR "adjuvant radiation therapy"[Title/Abstract] OR "postoperative radiotherapy"[Title/Abstract] OR "postoperative radiation therapy"[Title/Abstract] OR "post-operative radiotherapy"[Title/Abstract] OR "post-operative radiation therapy"[Title/Abstract] OR "postoperative chemoradiotherapy"[Title/Abstract] OR "postoperative chemoradiation"[Title/Abstract] OR "adjuvant chemoradiotherapy"[Title/Abstract] OR "adjuvant chemoradiation"[Title/Abstract] OR "chemoradiotherapy"[Title/Abstract] OR "chemoradiation"[Title/Abstract] OR "concurrent chemoradiotherapy"[Title/Abstract] OR "concurrent chemoradiation"[Title/Abstract] OR "de-escalation"[Title/Abstract] OR "deescalation"[Title/Abstract] OR "de-intensification"[Title/Abstract] OR "deintensification"[Title/Abstract] OR "dose reduction"[Title/Abstract] OR "radiation dose reduction"[Title/Abstract] OR "treatment sequencing"[Title/Abstract] OR "treatment sequence"[Title/Abstract] OR "multimodal treatment"[Title/Abstract] OR "multimodality treatment"[Title/Abstract] ) AND ( "head and neck cancer"[Title/Abstract] OR "head and neck cancers"[Title/Abstract] OR "head and neck tumor"[Title/Abstract] OR "head and neck tumors"[Title/Abstract] OR "head and neck tumour"[Title/Abstract] OR "head and neck tumours"[Title/Abstract] OR "head and neck neoplasm"[Title/Abstract] OR "head and neck neoplasms"[Title/Abstract] OR "head and neck squamous cell carcinoma"[Title/Abstract] OR "HNSCC"[Title/Abstract] OR "oropharyngeal cancer"[Title/Abstract] OR "oropharyngeal carcinoma"[Title/Abstract] OR "oropharyngeal squamous cell carcinoma"[Title/Abstract] OR "OPSCC"[Title/Abstract] OR "HPV-positive oropharyngeal cancer"[Title/Abstract] OR "HPV positive oropharyngeal cancer"[Title/Abstract] OR "human papillomavirus-positive oropharyngeal cancer"[Title/Abstract] OR "tonsil cancer"[Title/Abstract] OR "tonsillar cancer"[Title/Abstract] OR "base of tongue cancer"[Title/Abstract] OR "tongue base cancer"[Title/Abstract] OR "pharyngeal cancer"[Title/Abstract] OR "laryngeal cancer"[Title/Abstract] OR "hypopharyngeal cancer"[Title/Abstract] ) AND ("2010/01/01"[Date - Publication] : "2026/08/01"[Date - Publication]) AND english[Language] |

Note. MeSH terms were not used in the primary PubMed strategy; the PubMed search was based on Title/Abstract free-text terms.
